# Investigation of the solubility of protoporphyrin IX in aqueous and hydroalcoholic solvent systems

**DOI:** 10.3762/bjnano.16.89

**Published:** 2025-07-29

**Authors:** Michelly de Sá Matsuoka, Giovanna Carla Cadini Ruiz, Marcos Luciano Bruschi, Jéssica Bassi da Silva

**Affiliations:** 1 Department of Pharmacy, State University of Maringa, Maringa, PR, Brazil

**Keywords:** 5-aminolevulinic acid, photodynamic therapy, protoporphyrin IX, shake-flask, solubility test

## Abstract

Photodynamic therapy (PDT) is a non-invasive treatment involving a photosensitizer (PS), light source, and tissue oxygen. Protoporphyrin IX (PpIX) is commonly used as a PS due to its tumor-targeting properties and phototoxicity. However, the physicochemical properties of PpIX foster self-aggregation, which is a challenge for its incorporation into pharmaceutical formulations. This study aimed to evaluate the solubility of PpIX in distinct solvent systems to support the development of novel pharmaceutical formulations. The shake-flask method was employed, using purified water, 50% ethanol (EtOH50), 77% ethanol (EtOH77), absolute ethanol (EtOHabs), and polymeric systems containing 10% (w/w) poloxamer 407 (P407) in water, in EtOH50 or in EtOH77. Approximately 10 to 25 mg of PpIX was added to 25 mL of the solvent, and the solutions were stirred at 100 rpm, at 37 °C, for up to 96 h. The PpIX concentration was measured by using a validated method (*R* = 0.9973), with equilibrium reached within 30 min. The dissolution profiles of the micellar systems were also evaluated using the Korsmeyer–Peppas model with lag time (*t*_lag_), which indicated a Fickian diffusion mechanism, preceded by a thermodynamically driven accommodation stage of PpIX into the micelles. The solubility of PpIX ranged from 0.138 mg/mL in water to 0.593 mg/mL in water containing 10% (w/w) P407. The solubility of PpIX in EtOH50 and EtOH77 with 10% (w/w) P407 was 0.503 and 0.507 mg/mL, respectively, while EtOHabs yielded the lowest solubility among ethanolic solvents (0.179 mg/mL). These results indicate that water and EtOHabs are unsuitable solvents for PpIX, whereas the nanostructured systems containing P407 showed the greatest potential for future pharmaceutical applications, mainly the aqueous one because of its low toxicity considering topical preparations.

## Introduction

Photodynamic therapy (PDT) is a promising therapeutic modality that has raised keen interest in the treatment of various conditions including cancer and microbial infections [1–2]. This non-invasive treatment combines a photosensitizer (PS), a suitable light source, and molecular oxygen, generating reactive oxygen species that induce cellular damage [3–4].

Among the PSs used in PDT, protoporphyrin IX (PpIX) stands out as a natural precursor of hemoglobin and porphyrins, exhibiting low toxicity in its monomeric form [5]. When exposed to light, at a specific wavelength, PpIX absorbs energy and transfers it to molecular oxygen, generating reactive oxygen species (ROS). These ROS are highly toxic to cells, inducing oxidative damage in various biomolecules such as lipids, proteins and DNA, leading to cell death [6–7].

However, PpIX and other PSs have their hydrophobicity as drawback, which can lead to the formation of aggregates in aqueous solutions, compromising bioavailability and photodynamic activity [5]. The aggregation of the PS decreases its photoactive properties since, for its action, it must be in the monomeric form, in which its bioavailability and light absorption capacity will be increased [5].

To overcome this problem, the development of drug delivery systems, such as poloxamer-based ones, has played an important role on the delivery of dyes for PDT [8–10]. Poloxamers are triblock copolymers with thermosensitive properties, capable of forming micelles that encapsulate PpIX, increasing its solubility and facilitating its administration [7,11–12].

In this context, poloxamer 407 (P407) is notable for its biocompatibility and ability to form stable nanometric micelles [8,13–14]. Its amphiphilic nature allows for the self-assembly of monomers into micelles with a hydrophobic core of polypropylene oxide (PPO) and a hydrophilic corona of polyethylene oxide (PEO), creating an environment suitable for the encapsulation of hydrophobic drugs such as PpIX [15]. These micelles enhance drug solubility, protect against aggregation, and improve bioavailability in aqueous media [5,16].

Additionally, at high concentrations, P407 can transition into more complex nanostructured systems, such as hydrogels and lyotropic liquid crystals, providing additional control over drug release and stability. The thermosensitive and self-assembling properties of P407 make it suitable for advanced nanostructures drug delivery platforms, including polymeric microneedles and hydrogels, ensuring enhanced drug retention and permeation [15,17–18].

Therefore, the present study aimed to investigate the best solvent system for PpIX, in order to optimize the development of new drug delivery systems for PDT applications. Moreover, the potential of P407 as a feasible system for PpIX delivery was also evaluated in different media.

## Experimental

### Preparation of the systems

The systems were prepared at least 24 h before the test and used for up to seven days. Hydroalcoholic and polymeric systems were employed. Hydroalcoholic systems were prepared by mixing water and absolute ethanol (EtOHabs). The polymeric systems were developed by adding 10% (w/w) of P407 to each solvent (water, ethanol 50% and 77% v/v), both previously weighed. The polymeric systems were refrigerated for 24 h and homogenized for 30 min prior to the addition of PpIX [19–20].

### Equilibrium solubility

The solubility of PpIX disodium salt was determined using the shake-flask method. Aliquots of 10 to 25 mg of PpIX were added to 25 mL of aqueous, hydroalcoholic (EtOH50, EtOH77, EtOHAbs), or polymeric systems. Experiments were conducted in triplicate. The resulting systems exhibited concentrations of 0.4 mg/mL (EtOH50, EtOH77, and water) and 1 mg/mL (EtOHAbs). For the systems containing P407 in EtOH50, EtOH77, and water, a PpIX concentration of 0.4 mg/mL was used. The flasks were sealed, protected from light, and maintained under agitation at 37 ± 1 °C and 100 rpm. Aliquots of 50 to 500 µL were collected at time intervals up to 96 h, diluted in 77% ethanol (v/v), filtered through a 0.45 µm membrane, and quantified by spectrophotometry at λ = 400 nm. Quantification was performed using a previously validated method, yielding a correlation coefficient *R* = 0.9973 [20–21].

### Transmission electron microscopy

Transmission electron microscopy (TEM) analysis was carried out using a JEM-1400 microscope (JEOL, Tokyo, Japan) with an accelerating voltage of 100 kV. The samples were diluted 50 times and negatively stained with a 2% (w/v) uranyl acetate solution before imaging. To investigate micelle formation, the samples were prepared at 37 °C. Micelle size measurements obtained by TEM were reported as the mean (± standard deviation; SD), based on the analysis of 250 micelles per system using ImageJ software [17].

### Statistical analysis

Data are presented as mean ± SD. Statistical analysis was performed using Microsoft Office Excel^®^, version 2312, conducting Student’s t-test one-way analysis of variance (ANOVA), followed by Tukey's post-hoc test. Results were considered statistically significant when *p* < 0.05, with a 95% confidence interval.

## Results and Discussion

**Figure 1:**
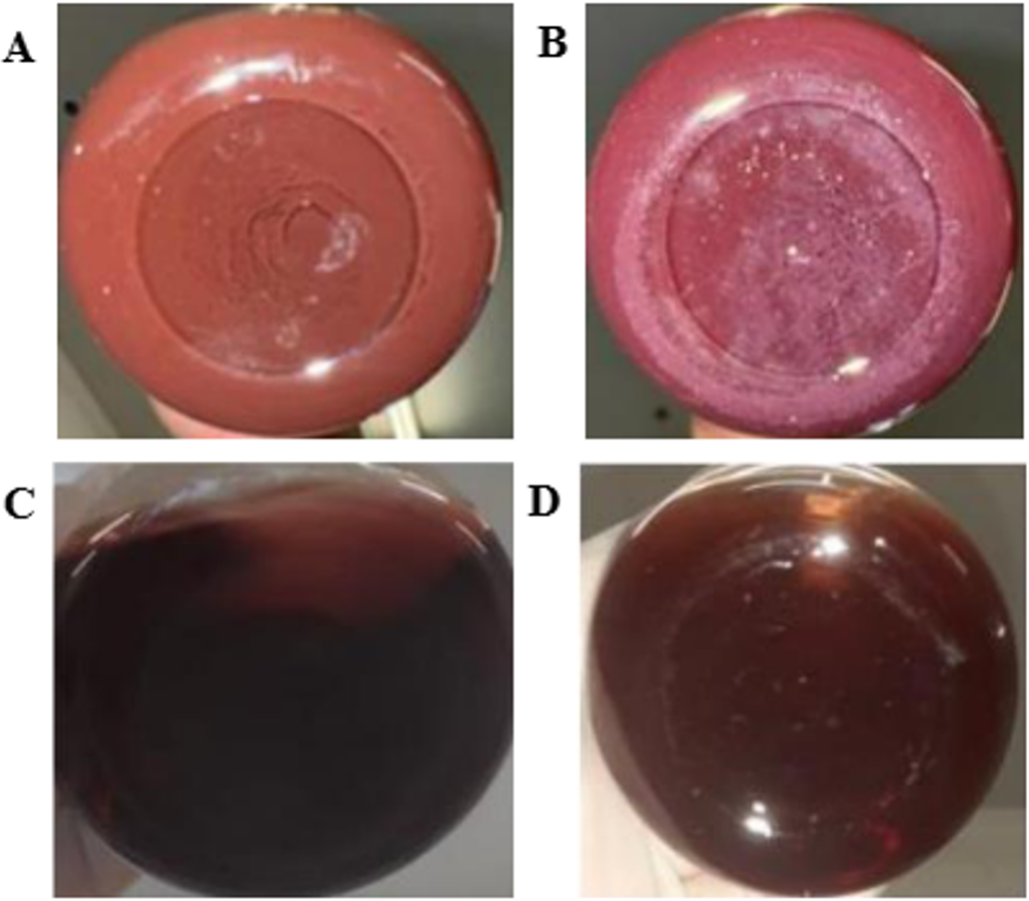
Macroscopic characteristics of solubilized systems: (A) water (0.4 mg/mL), (B) absolute ethanol (1 mg/mL), (C) 50% (v/v) ethanol (0.4 mg/mL), and (D) 50% (v/v) ethanol with 10% (w/w) P407 (0.4 mg/mL).

Systems containing water (0.4 mg/mL PpIX) and EtOHabs (1 mg/mL) displayed persistent visible precipitates from 30 min to 48 h, indicative of PpIX saturation in these solvents. As illustrated in Figure 1, while EtOH50 and EtOH77 effectively solubilized PpIX up to 0.4 mg/mL, water and EtOHabs rapidly reached saturation, demonstrating instability. Solubility equilibrium was attained within approximately 4 h for EtOH50 and EtOH77, whereas for water and EtOHabs, equilibrium was established within 30 min, followed by subsequent degradation or aggregation.

The addition of P407 significantly enhanced PpIX solubilization, particularly in aqueous systems, with no significant differences observed between water and ethanol at varying concentrations in the presence of P407. Figure 1A and Figure 1B highlight the turbidity observed in these samples, whereas samples shown in Figure 1C and Figure 1D appear clear. The sample displayed in Figure 1D was the most soluble and transparent, demonstrating the improved system performance in the presence of P407.

Although Franco [5] reported high solubility of PpIX in aqueous systems, the findings observed at 0.4 mg/mL PpIX are not in line. While spectrophotometric readings indicated high solubility, the visual inspection revealed turbidity, suggesting incomplete dissolution of the photosensitizer. This discrepancy may be attributed to the filtration step performed prior to UV–vis analysis, which intended to mitigate the limitation of the spectrophotometric techniques in the presence of aggregated species. In good agreement, similar discrepancies have been reported in the literature, such as in the study by da Silva Gonçalves and collaborators [22], where spectroscopic estimations of solubility were later contradicted by particle size distribution analysis.

**Figure 2:**
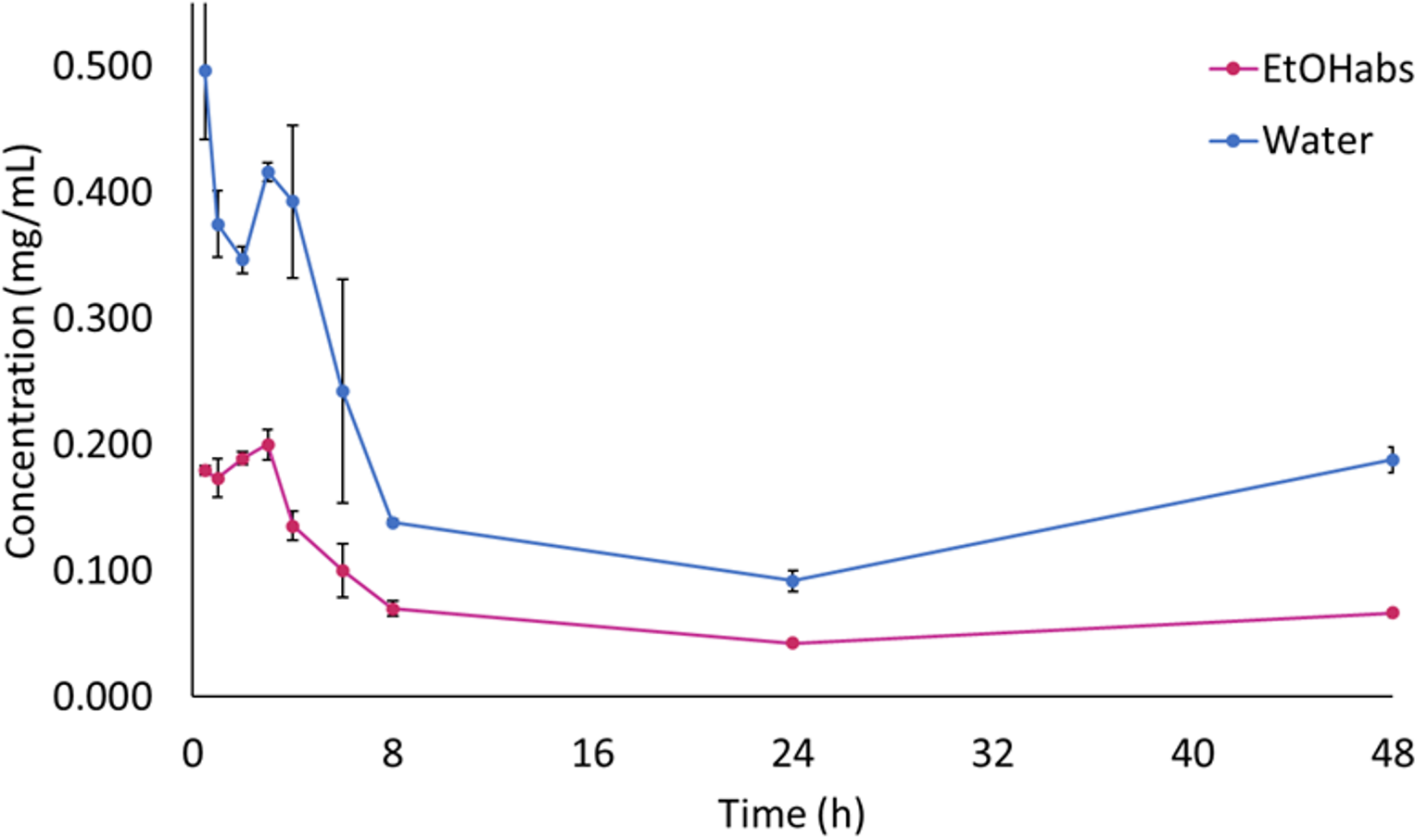
Solubilization kinetic curve of PpIX in water and EtOHabs. Error bars represent one standard deviation (*n* = 3).

Figure 2 shows the solubility behavior of PpIX in water and EtOHabs, with mean concentrations of 0.497 ± 0.054 mg/mL in water, and 0.179 ± 0.004 mg/mL in EtOHabs.

**Figure 3:**
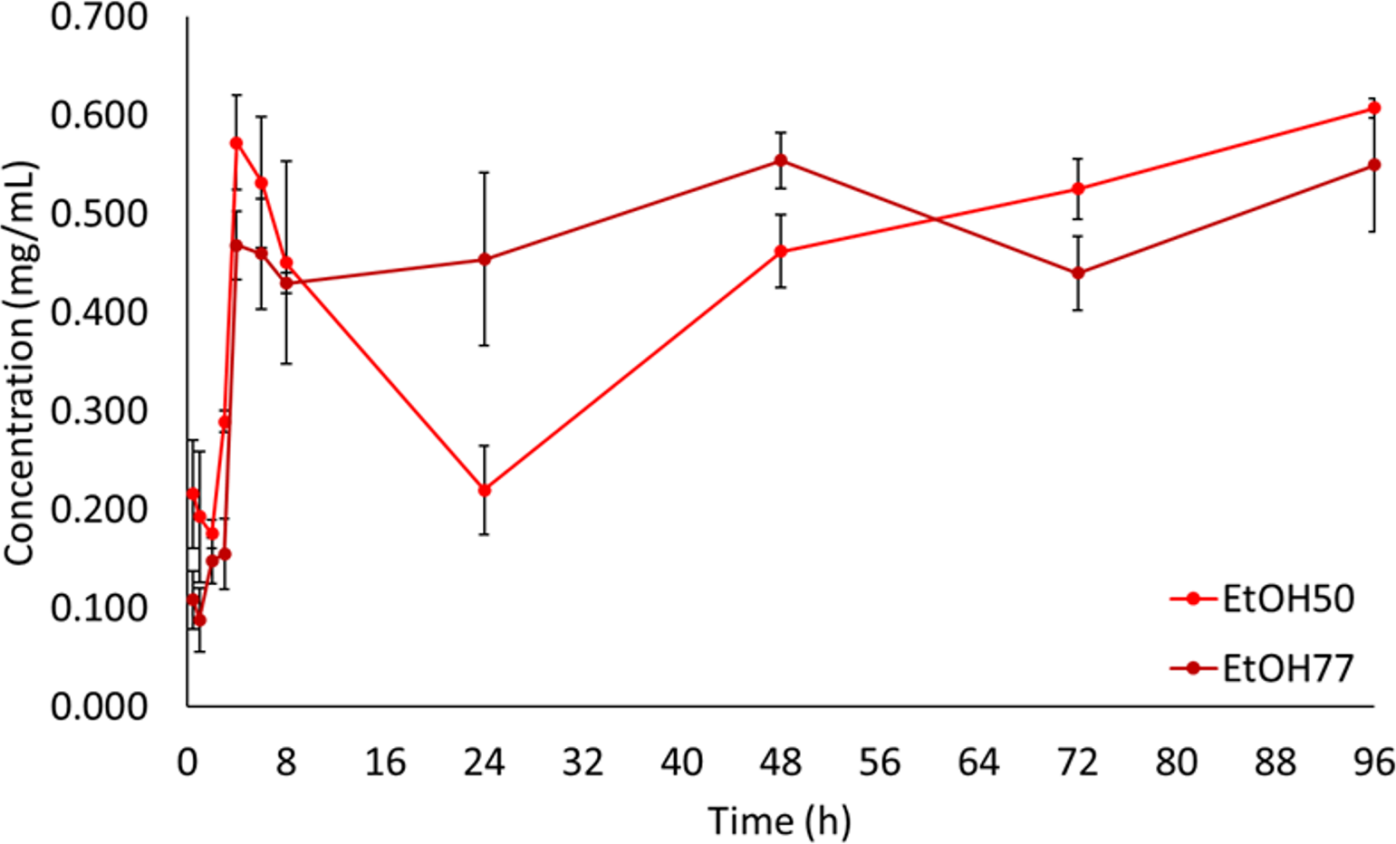
Solubilization kinetic curve of PpIX in EtOH50 and EtOH77 solvents. Error bars represent one standard deviation (*n* = 3).

In contrast, the systems containing 0.4 mg/mL of PpIX in EtOH50 and EtOH77 remained clear and free of undissolved solids for up to 96 h, confirming the complete solubilization without saturation. Figure 3 illustrates PpIX concentration during the solubilization period in EtOH50 and EtOH77. The average concentration over the first 8 h of analysis (stabilization period) was 0.450 ± 0.103 mg/mL for EtOH50 and 0.430 ± 0.010 mg/mL for EtOH77. These values align with findings by da Silva Gonçalves and collaborators [22], who discussed the modification of porphyrin derivatives to enhance solubility, and Rossin and colleagues [2], who reported similar solubility ranges for porphyrin-based compounds in hydroalcoholic systems.

Overall, the results indicated that while EtOH50 and EtOH77 effectively dissolve PpIX at concentrations up to 0.4 mg/mL, water and EtOHabs rapidly reach saturation and exhibit instability. The solubility equilibrium was achieved after approximately 4 h for EtOH50 and EtOH77, whereas for water and EtOHabs, the equilibrium was established within 30 min, followed by degradation or aggregation. These results support the observations made by Tima and collaborators [11], who found that ethanol–water mixtures offer an improved balance between solubility and stability for hydrophobic compounds.

**Figure 4:**
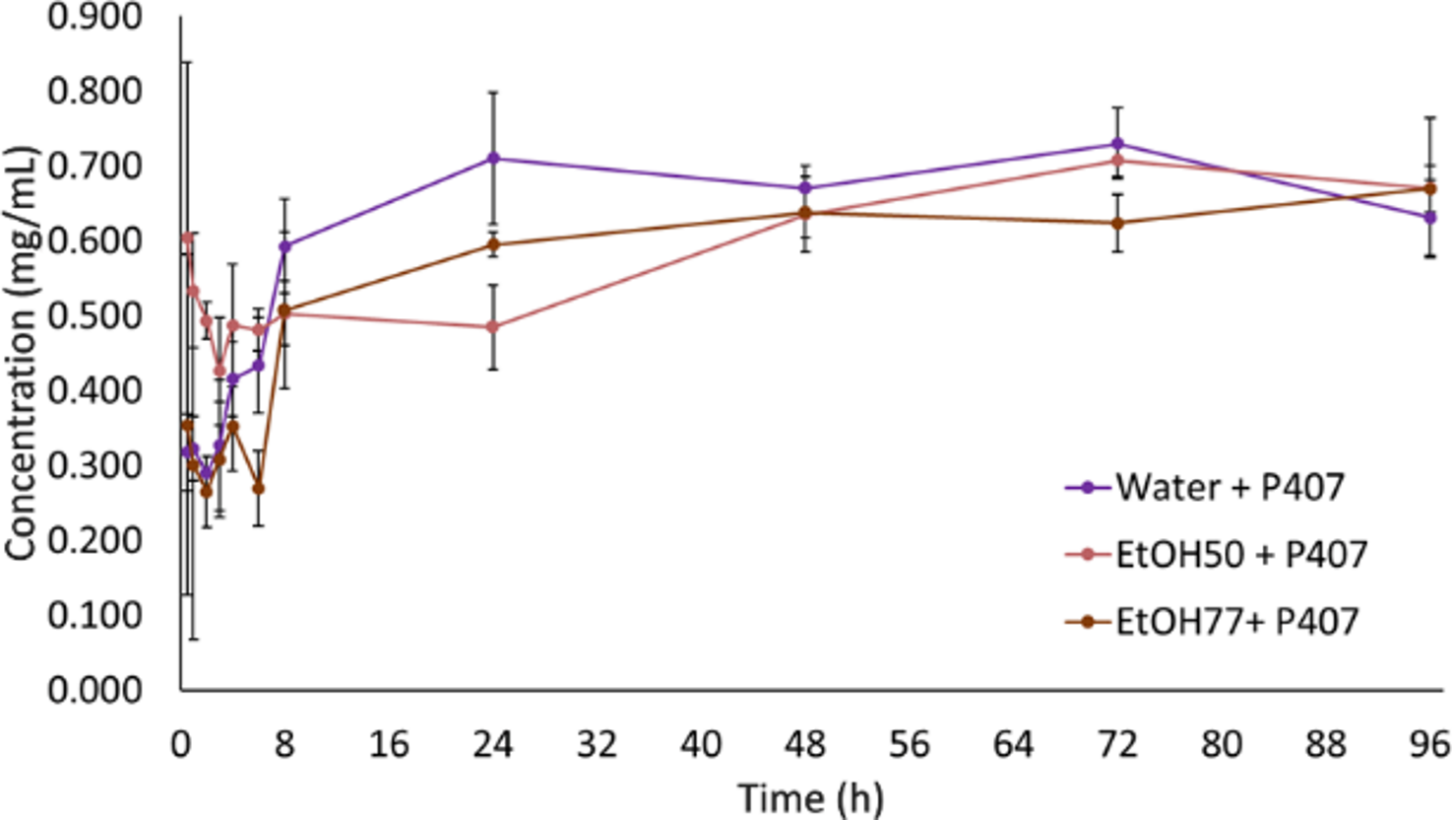
Kinetic curves of PpIX in polymeric systems. Error bars represent one standard deviation (*n* = 3).

In systems containing 10% (w/w) P407 with 0.4 mg/mL PpIX (EtOH50, EtOH77, and water), the complete solubilization was achieved regardless of the solvent used. Figure 4 shows the solubilization kinetics in the polymeric systems. The average concentrations after 8 h were 0.593 ± 0.063 mg/mL for water-based systems, 0.503 ± 0.043 mg/mL for EtOH50, and 0.507 ± 0.104 mg/mL for EtOH77, which is consistent with previous reports on micellar stabilization of hydrophobic drugs [16].

These systems remained clear and free of precipitates for up to 96 h, suggesting the formation of a stable micellar solution. The presence of P407 enhanced PpIX dissolution, even in water, which otherwise exhibited some limitations. Mathematical models were employed to better understand and simplify the complex dissolution behavior of the micellar systems. Several models, including zero-order, first-order, Higuchi, and Korsmeyer–Peppas were evaluated for both conventional and micellar systems. However, most of them could not fit the results. The best fit was obtained by using the Korsmeyer–Peppas model with lag time (*t*_lag_), only for the systems containing P407 (Table 1).

**Table 1:** Mathematical modeling of the solubilization curves of PpIX dissolution in micellar systems using the Korsmeyer–Peppas model with lag time (*t*_lag_).

PpIX in polymeric systems	Korsmeyer–Peppas with *t*_lag_		

	k (h^−n^)	n	*R*

water + P407	82.772	0.174	0.9007
EtOH50 + P407	124.062	0.042	0.5355
EtOH77 + P407	75.728	0.164	0.8935

The lag time parameter in the model reflects the initial phase of micelle formation and the subsequent accommodation of PpIX into the micellar core, which precedes the diffusion-driven dissolution process [23]. Among the systems evaluated, water + P407 and EtOH77 + P407 exhibited the highest correlation coefficients (*R* = 0.9007 and 0.8935, respectively), indicating a strong fit and supporting the hypothesis of micelle-mediated stabilization of PpIX in these environments.

The release exponent (n) values obtained were all smaller than 0.45, which is characteristic of Fickian diffusion. This suggests that the dissolution mechanism is mainly governed by simple diffusion, without significant swelling or erosion of the polymeric matrix [8], which is commonly observed in hydrophilic polymeric systems, as those produced by P407 [24]. In this context, the drug molecules diffuse through the micellar network driven by concentration gradients, while the micellar structure remains intact throughout the dissolution process [25]. The kinetic constant (k) was elevated for all the systems, although polymer, temperature, and the solvent properties may influence this parameter.

Overall, the water + P407 system demonstrated the most favorable behavior, as no micellar disintegration was observed, unlike the systems containing ethanol. These findings are consistent with other experimental results, reinforcing the stability and solubilizing potential of aqueous micellar systems for PpIX [8].

To improve the assessment of solubility, complementary methods may be performed together, such as dynamic light scattering [26] and turbidimetry [27]. Furthermore, TEM analysis can provide a more accurate understanding of the incorporation of PpIX into the micellar systems, overcoming the limitations associated with spectrophotometric estimations. These limitations include potential overestimation of solubility due to light scattering by aggregates, turbidity-related interference, and the inability to distinguish between molecularly dissolved and aggregated species [28].

**Figure 5:**
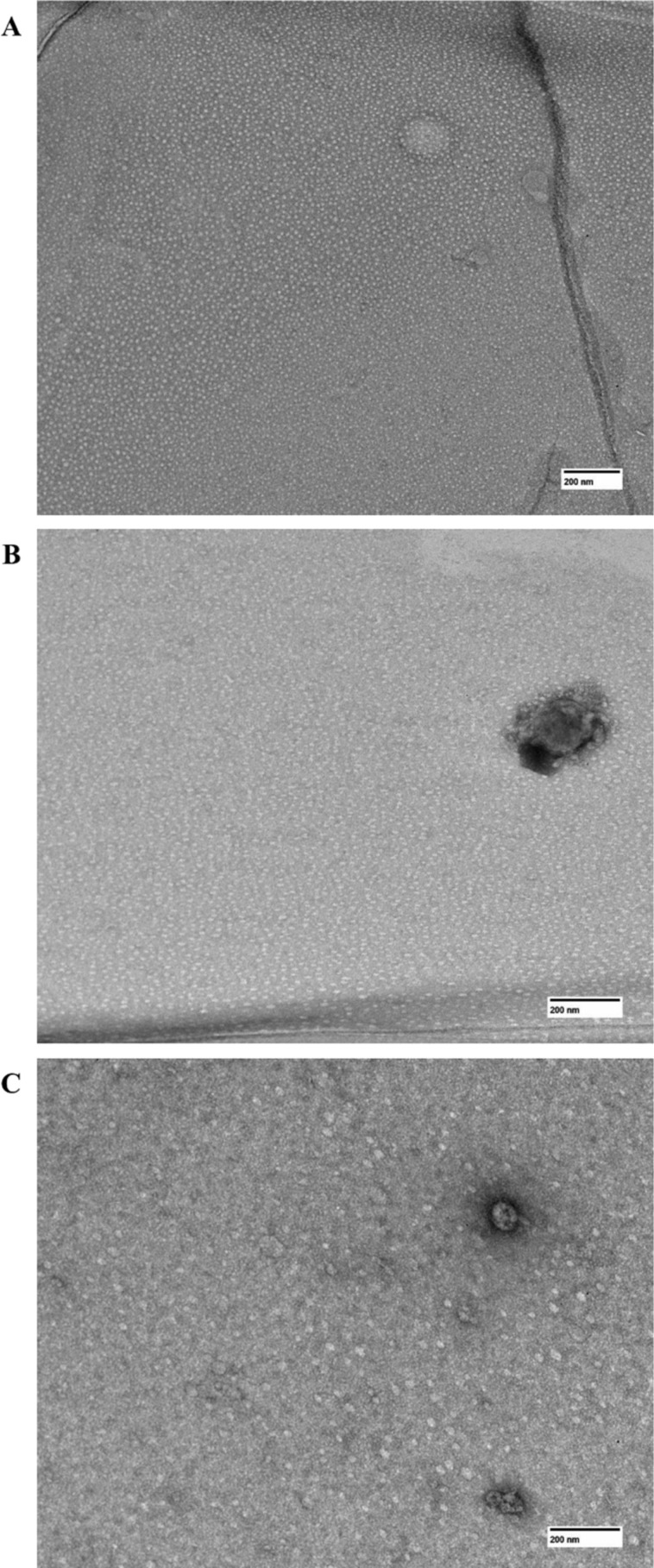
TEM images of PpIX in polymeric systems comprising 10% (w/w) poloxamer 407 in purified water (A), EtOH50 (B) and EtOH77 (C). The images are at a scale of 200 nm.

Therefore, TEM micrographs of PpIX samples in different media containing P407 (water, EtOH50, and EtOH77) are displayed in Figure 5. It was possible to observe significant variations in micelle size and morphology. While in the aqueous system well-defined circular micelles are observed with an average diameter of 11.81 ± 1.71 nm, in EtOH50 and EtOH77 the samples exhibited progressively large micelles (13.56 ± 3.24 nm and 14.77 ± 3.81 nm, respectively). This suggests that ethanol, due to its dissolution properties, may partially disrupt the micelles, resulting in larger and less homogeneous structures [29].

The disrupted micelles formed in alcoholic solution may exhibit limited effectiveness in encapsulating PpIX for drug delivery in biological systems. Moreover, the small size of the micelles observed in water tend to exhibit improved permeability, which may present significant implications for the application of PpIX in biological systems [17].

## Conclusion

This study demonstrated that hydroalcoholic solutions and P407-based systems effectively solubilized PpIX at 0.4 mg/mL yielding clear and stable solutions, in contrast to the low solubility and turbidity observed in aqueous and absolute ethanol systems. P407 significantly enhanced PpIX solubility, with no significant differences among water-based, 50% v/v ethanol, and 77% v/v ethanol polymeric systems. Considering the potential toxicity and the challenges of hydroalcoholic formulations, the aqueous P407 system, with its gelling and thermoresponsive properties, stands out as the most promising approach for further pharmaceutical applications. TEM micrographs revealed that PpIX micelles in water formed smaller and more uniform structures, suggesting improved drug load ability and permeability compared to alcoholic solutions. Moreover, dissolution kinetics, modeled using the Korsmeyer–Peppas equation with lag time (*t*_lag_), indicated a Fickian diffusion mechanism occuring after the initial phase of micelle formation and subsequent accommodation of PpIX within the micellar core of the P407-based systems. These results reinforce the structural stability and controlled-release potential of the micellar formulations, particularly the aqueous one, strengthening their suitability for topical drug delivery strategies.

## Data Availability

Data generated and analyzed during this study is available from the corresponding author upon reasonable request.
